# DAMPs and Innate Immune Training

**DOI:** 10.3389/fimmu.2021.699563

**Published:** 2021-10-22

**Authors:** Elisa Jentho, Sebastian Weis

**Affiliations:** ^1^ Instituto Gulbenkian de Ciência, Inflammation Laboratory, Oeiras, Portugal; ^2^ Department of Anesthesiology and Intensive Care Medicine, Jena University Hospital, Friedrich-Schiller-University, Jena, Germany; ^3^ Institute for Infectious Disease and Infection Control, Jena University Hospital, Friedrich-Schiller-University, Jena, Germany

**Keywords:** DAMP, trained innate immunity, heme, vimentin, oxLDL

## Abstract

The ability to remember a previous encounter with pathogens was long thought to be a key feature of the adaptive immune system enabling the host to mount a faster, more specific and more effective immune response upon the reencounter, reducing the severity of infectious diseases. Over the last 15 years, an increasing amount of evidence has accumulated showing that the innate immune system also has features of a memory. In contrast to the memory of adaptive immunity, innate immune memory is mediated by restructuration of the active chromatin landscape and imprinted by persisting adaptations of myelopoiesis. While originally described to occur in response to pathogen-associated molecular patterns, recent data indicate that host-derived damage-associated molecular patterns, *i.e.* alarmins, can also induce an innate immune memory. Potentially this is mediated by the same pattern recognition receptors and downstream signaling transduction pathways responsible for pathogen-associated innate immune training. Here, we summarize the available experimental data underlying innate immune memory in response to damage-associated molecular patterns. Further, we expound that trained immunity is a general component of innate immunity and outline several open questions for the rising field of pathogen-independent trained immunity.

## Introduction

Monocytes and macrophages (Mφ) are professional phagocytotic cells (1), a feature first described by Elie Metchnikoff almost 150 years ago (2). Circulating monocytes originate from the bone marrow and can differentiate into monocyte-derived Mφ and dendritic cells upon stimulation (3, 4) and subsequently elicit a robust inflammatory response, which includes the secretion of cytokines. This qualifies these cells as initiators of inflammation and places them in the first line of defense against invading pathogens (3, 5). In contrast, tissue resident Mφ, derived from the yolk sac or the fetal liver, are thought to regulate organ development and homeostasis as well as to control resolution of inflammation (5, 6). However, this is not a fixed dichotomy and under specific conditions, monocyte-derived Mφ can also acquire a phenotype that promotes homeostasis and tissue repair similar to tissue-resident Mφ (5).

In contrast to adaptive immunity that develops antigen-specific memory, the cellular components of the innate immune system, including monocytes and Mφ, were long thought not to remember previous stimulation. Instead after a transient phase of recovery, it was assumed that they would react in a similar and repetitive way to inflammatory stimuli (7).

## Memory of the Innate Immune System

The above-described perspective was challenged during the last 15 years by several independent discoveries that showed persistent histone modifications in Mφ in response to the bacterial cell-wall component Lipopolysaccharide (LPS), the fungal cell wall component β-1,3-D-glucan among others (8–10). The phenomenon of acquired and persistent alterations of innate immune responses was coined as innate immune memory and presents typically as tolerance, referring to a reduced response or trained immunity (TRIM), referring to an enhanced response upon restimulation (10).

**Figure 1:**
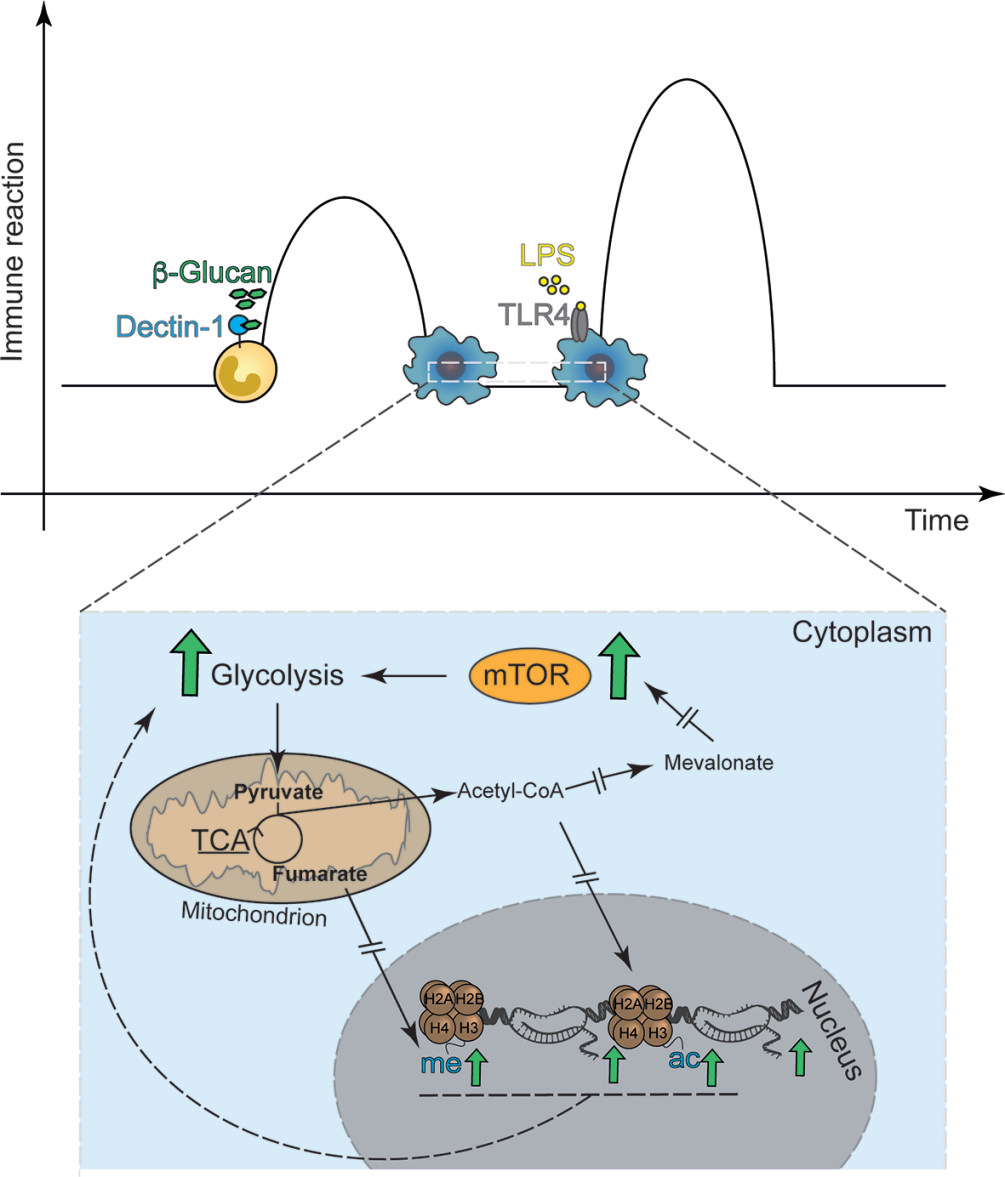
Classical *in vitro* model of trained immunity. Trained immunity describes a functional, metabolic and epigenetic adaptation of innate immune cells to previous stimuli with ensuing increased immune response, i.e. cytokine release, to secondary stimulation. **(A)** The classical model applies the Dectin-1 agonist β-1,3-D Glucan as the first stimulus and the TLR-4 agonist LPS as the second stimulus. **(B)** The basis for β-1,3-D Glucan induced trained immunity are metabolic adaptations, including the mTOR signal-transduction enhanced glycolysis. Interrupted errors indicate that many more proteins are involved in the signaling cascade, which are not depicted in the figure.

The first observation that LPS-mediated Toll-like receptor (TLR)-signaling induced gene-specific chromatin modifications were made by Foster et al. when aiming to understand immunotolerance (8). The authors revealed a set of gene-specific chromatin modifications that are associated with gene silencing or enhanced response to re-exposure (8). In addition, it was established that a subset of genes could be persistently tolerized while others remained unaffected or even had enhanced transcription, the latter set being described as non-tolerizeable genes. Subsequent work by other groups revealed that the fungal cell wall component β-1,3-D-glucan and other inflammatory stimuli can also induce specific and persistent modifications of histone acetylation and methylation, underlying a long-term modulation of the innate immune response (**Figure 1**) (9, 11). Both phenomena share common characteristics, *e.g.*, exposure to a given stimuli ensues long-term modulation of the innate immune response to that same or related stimuli and are associated with long-term modification of gene transcription (8, 9). TRIM *in vivo* and *in vitro* was first demonstrated using the fungal cell wall component of *Candida albicans* β-1,3-D-glucan, a *bona fide* pathogen-associated molecular patterns (PAMP) or the Bacille-Calmette Guerin (BCG) the live-bacteria tuberculosis vaccine (9, 11–13). These molecules commonly use the mechanistic Target of Rapamycin (mTOR) pathway to induce TRIM to activate specific downstream metabolic adaptations (14, 15). In fact, in myeloid cells, TRIM relies on alterations of the cellular energy metabolism involving glycolysis, itaconate synthesis, glutaminolysis and fumarate metabolism (16–18). While the increase in glycolysis seems to be a shared mechanism between the different trained immunity inducers, the regulation of OXPHOS, *e.g.* repression or activation, appears to be stimulus-specific. Exposure to β-glucan also leads to increased abundance of histone marks H3K4me3 and H3K27ac especially at promotors of genes encoding proteins regulating glycolysis. In addition, glutaminolysis, which is activated during trained immunity fuels the TCA cycle, accumulating specific metabolites, such as fumarate, which even further increases histone marks at H3K4me3 and H3K27ac. Fumarate, can also directly inhibit the activity of histone demethylases (17) placing it at a central hub to for the metabolic control of β-glucan induced TRIM. Consistently, inhibition of glycolysis and glutaminolysis reduced these histone marks at the promotors of IL6 and TNFA. In addition, increased amounts of mevalonate, a metabolite involved in cholesterol synthesis (18, 19) upregulate the IGF-I signaling pathway, which in turn promotes the activation of the mTOR pathway and glycolysis. This results most likely in the accumulation of acetyl-CoA an important donor for acetyl groups for histone acetylation (20). This data is suggestive for a strong interaction of metabolic and inflammatory pathways, underling trained immunity (14, 17, 18, 21).

TRIM can be induced *in vivo* in mice *via* a mechanism that is at least partially based on modified hematopoiesis, favoring myelopoiesis and potentially increasing host resistance to infection (22–25).

As described before, LPS tolerance is associated with specific gene silencing or enhanced response to re-exposure (8). It is conceivable that the same would occur in TRIM, *i.e.* that innate immune training includes not only trainable but also non-trainable genes probably including tolerizeable genes. This raises the possibility that training, and tolerance are not unrelated phenomena but rather dependent on the re-writing of gene activation and repression programs *via* specific stimuli-induced signaling pathways to specify the reaction pattern of the innate immune cells. This notion is supported by the finding that LPS-induced histone modifications can be reversed at specific loci by different secondary stimuli (26). We speculated that this regulation underlies why training and tolerance are detrimental in some and protective in other models (14, 22). However, this needs further experimental evidence, which is why we use the TRIM terminology to describe memory that induces enhanced responses.

## Damage and Danger

The prevailing concept since the 1950s that the immune system evolved to distinguish self from non-self was challenged in a seminal essay by Polly Matzinger (27). She proposed that the immune system does not exclusively differentiate between foreign and self but instead evolved to detect cues indicating danger. Matzinger´s danger theory was primarily intended to understand T-cell biology. This theory contains specifically the idea that professional antigen presenting cells are activated “in the presence of tissue destruction” (27). Whether this is a completely novel approach or a reappraisal of earlier thoughts is not the topic of this review (28). According to Matzinger, immune cells are primarily made for sensing detecting danger and only sense invading microorganisms for the reason that infections typically are associated with danger, in the form of cellular stress and damage (27, 29). In the classical concept of immune recognition, DAMPs would in fact be considered as “self”. However, it has become clear that certain host-derived molecules can activate innate immunity and induce an inflammatory response regardless whether they are triggered by infection or by sterile inflammation (30). These molecules have been designated as damage or danger-associated molecular pattern (DAMP) and are also referred to as alarmins by some authors. An overview of the different terminology is shown in **Table 1**.

**Table 1:** Definitions of PAMP, DAMP and defined sub entities.

Molecule	Abbreviation	Characteristics	Examples
Pathogen-associated molecular pattern (PAMP)*	PAMP	Conserved microbial molecules which are sensed by pattern recognition receptors (31).	Lipopolysaccharid (LPS) (32)
			β-Glucan (33)
**Damage-associated molecular pattern**	DAMP	Any molecule which is exposed during, after, or because of disrupted cellular homeostasis such as damage or injury (34)	HMGB-1(35)
			ATP (36)
**Or earlier**			Heme (37)
**Danger-associated molecular pattern**			
**Alarmin**		Endogenous molecules, released by damaged cells, during cell death and degranulation. Constitutively expressed.	Vimentin (38)
		Provoke chemotactic and immune activating reactions by interacting with PRR (39).	Defensins, Cathelicidin, Eosinophil-derived neurotoxin (40)
			Heme (37)
**Nematode-associated molecular patterns (only specifically described for plants)**	NAMP	Nematode-derived molecules that initiate an early immune response/defense in plants.	Ascarosides (nematode pheromones) and unidentified molecules released from plant pathogenic nematodes (41).
		Receptors unknown	
**Lifestyle-associated molecular patterns**	LAMP	Non-PAMP, non-DAMP molecules that induce an inflammatory response.	Cholesterol; Monosodium urate; Oxidized LDL (42)
		Cannot be cleared. Persistence leads to chronic inflammation	
**inducible DAMP**	iDAMP	Inflammation-inducing molecules actively produced or modified during cell death. Proposed to reflect cellular stress response and cell death pathways	IL1b, IL18 Heat shock proteins (43)
**constitutive DAMP**	cDAMP	Inflammation-inducing molecules that are already present intracellularily before cell death/stress and are released by dying cells (43).	HMGB-1
			mtDNA
			ATP
			Heme

*Some authors preferentially refer to PAMPs as same molecule as microbe-associated molecular patterns as also commensal bacteria express these genes without exerting pathologies (42). Other authors have used the same abbreviation to define a subset of DAMP subset as metabolism-associated molecular pattern (44). We consider this nomination confusing and restrain from using it in this review.

Overall, DAMPs are a rather heterogenous group of molecules with shared common features. They are *a*) host-derived and not pathogen- or environment-derived and *b*) induce an innate immune response. In order to acknowledge their heterogeneity, DAMPs have recently been further subclassified as continuous DAMPs (cDAMPs), inducible DAMPs (iDAMPs) (**Table 1**) (42, 43). In this classification, cDAMPs are intracellular molecules that are not present in the circulation under non-pathological conditions and are set-free without modifications upon cellular damage. iDAMPs are secreted and/or induced molecules, released from dying cells and have been proposed to reflect various stress and damage pathways activated during stress (43). DAMPs are heterogenous in their origin and function. Yet, they induce a rather homogenous sterile inflammation that equally involves cytokine release, neutrophil recruitment and the induction of T-cell immunity equally to the response elicited by PAMPs (30). The recently classified group of lifestyles—associated molecular patterns (LAMP) consist of molecules increased with western lifestyle hat induce a sterile inflammation. These are distinct from DAMPs as they cannot be cleared, and if persistent, lead to a chronic inflammation. This group includes cholesterol, monosodium urate or oxidized LDL and others (42) (**Table 2**).

**Table 2:** DAMPs for which innate immune training has been shown.

Molecule	Applied Models	Outcome	Pathway	REF
**Heme**	human/murine monocytes/ Mϕ, LPS shock, Polymicrobial sepsis, *in vitro* and *in vivo*	Dual role depending on the second stimulus.	Syk/JNK	(22)
**Vimentin**	HMGB-1-treated murine Mϕ	Increased release of pro-inflammatory cytokines	mTOR	(45)
**oxLDL**	Human monotyes/ Mϕ	Increased release of inflammatory cytokines	Endothelial cells: TLR2 mTOR/Hif1α	(46)
		Endothelial cells: Cytokines and expression ICAM1, VCAM1, E-selectin		(47–49)

## Conserved Pattern Recognition to Damp and Pamp

Monocytes and Mφ express different sets of pattern recognition receptors (PRRs) that bind to PAMPs (50) and DAMPs (51–53). There are four distinct classes of PRR that are identified so far: Toll-like receptors (TLR), nucleotide-binding oligomerization domain (NOD)- Leucine-rich repeats (LRR)-containing receptors (NLR), retinoic acid-inducible gene 1 (RIG-1) -like receptors (RLR), and the C-type lectin receptors (CLR) (54). Binding of PAMPs and DAMPs by PRRs triggers distinct signaling transduction pathways which elicit the expression of immunomodulatory molecules, *e.g*. cytokines, indispensable for an appropriate immune reaction against an exogenous or endogenous threat.

TRIM can be induced by different classes of PRRs, as illustrated by β-glucan, which binds to the C-type lectin receptor Dectin-1 activating the noncanonical Raf-1 pathway signaling (12). So far only one intracellular PRR has been identified to result in TRIM upon engagement, namely, NOD-2/Rip2 in response to BCG (11). This is fundamentally different to the immunological tolerance which involves TLR-4 activation and the NF-κB/MAPK pathway (55). We here posit that DAMP-induced TRIM shall not involve cytoplasmatic PRR such as NLR or RIG-1. This is because by definition, a DAMP is an endogenous, *i.e.* cytoplasmatic or nucleic, molecule that is released in the circulation and then bound by extracellular receptors, with the potential to be endocytosed after ligand-binding (56). In contrast, cell intrinsic stress responses mount conserved stress-control pathways that prevent tissue damage (57). the release of DAMPs and as a consequence also the ensuing activation of the immunes system.

## Damps as Trainers

Compared to PAMP-induced TRIM, DAMP-induced TRIM is less well studied and understood. Five years ago, Crisan et al. had speculated on the existence on DAMP-induced trained immunity and summarized concepts and early data (58). During the last years, increasing amounts of evidence shows that endogenous molecules promote in fact TRIM include the iron-containing tetrapyrrole heme (22), the intermediate filament vimentin (45), oxidized low-density-lipoproteins (oxLDL) (46) and the mineralocorticoid aldosterone (59). An overview of the studies is provided in **Table 2**. As aldosterone is a hormone and not considered a DAMP, it will not be discussed further in this review.

Both heme and vimentin are alarmins that can activate PRR signaling either by TLR-4 or Dectin-1, respectively (37, 38). OxLDLs are a heterogenous group of molecules that, depending on their oxidation status, bind to different PRR. Minimally modified LDL can directly bind to cluster of differentiation (CD)14, TLR-2 and -4 triggering immune activation (48, 60, 61). Further oxidized OxLDL is recognized by a family of scavenger receptors including the lectin-like oxidized low-density lipoprotein receptor-1 (LOX-1), CD36 and the scavenger receptor class B type I (SR-BI) (62). In the following paragraphs we will briefly summarize the findings for the individual TRIM-promoting DAMPs.

### Heme

Heme is a tetrapyrrole with a central iron atom found in hemoglobin and other hemoproteins. The reactive central iron, which is responsible for the biological heme functions can reversibly change its oxidation state from ferric (Fe^3+^) to ferrous (Fe^2+^) to accept or donate electrons, respectively. This reactive core makes heme not only an indispensable molecule for many physiological processes, but it also bears the risk for cytotoxicity when unbound to proteins. As such, heme is able to oxidize lipids and proteins and can induce DNA damage (63, 64). Additionally, heme can promote the generation of free radicals *e.g*. when reacting with other organic hydroperoxides, further imposing cellular damage (63, 64). Under homeostatic conditions heme production and degradation are tightly controlled processes. Following hemolysis or tissue damage, heme is passively released into the circulation. There it is bound non-covalently by serum scavenger proteins and taken up by Mφ (65–70). As far as we know now, there is no active heme export.

With increasing concentrations, the buffering capacity of serum protein becomes exhausted resulting in the accumulation of cell-free, ‘labile’ heme in the plasma (70, 71). This contributes critically to the pathogenesis of severe acute infectious disease, as demonstrated for malaria (72) and for bacterial sepsis (73–75). Labile heme is a pro-type alarmin that is sensed by TLR-4 but also activates the spleen tyrosine kinase (Syk) pathway both inducing cytokine expression, including the cytokines IL-6 and pro-IL-1β in innate immune cells (37, 76). As heme synergized with LPS with regards to cytokine release, it was assumed that heme would bind to a distinct pocket of TLR-4 and specifically induced MyD88 signaling (77). How heme triggers Syk signaling is currently unknown (37, 76, 78). We have recently described that heme is a potent inducer of TRIM which is mediated by the activation of Syk (22). In contrast to other TRIM inducers this is independent of mTOR. However, *in vivo* heme training causes comparable expansion of myeloid primed long-term hematopoietic stem cells as seen in PAMP-induced TRIM (19, 79).

In line with the above, Schrum et al. identified that damaged red blood cells and hemozoin crystals, as a result of a malaria-inducing *Plasmodium falciparum* infection, induce TRIM in primary monocytes *in vitro* (80). *Plasmodium* spp. replicate in erythrocytes and regularly disrupt their cell membrane to be released into the circulation which is accompanied by the release of the *Plasmodium -*metabolic byproduct hemozoin (81, 82). This study perceives the *Plasmodium* induced TRIM to be a result of PAMPs and does not consider that damaged red blood cells as well as hemozoin are major sources of labile heme. Together with the findings of Jentho et al. (22), TRIM seems to be an inherent component of innate immune cells considering the wide range of infections associated to release of labile heme. Given the human-pathogen co-evolution especially with *Plasmodium* spp. these studies raise the question what kind of evolutionary advantage is achieved by inducing TRIM.

### Oxidized LDL

Oxidized LDL encompasses a number of different particles such as protein and fatty acids with varying oxidation states (83, 84). Application of *in vitro* oxLDL induces TRIM in Mφ, as well as in non-hematopoietic lineage cells such as endothelial cells and human coronary smooth muscle cells (46, 48, 49). OxLDL bind to a family of scavenger receptors that include CD36 (62) which in turn can activate TLR-4 and TLR-6 signaling (85). Mechanistically, as seen in β-glucan, oxLDL-TRIM is associated with mTOR signaling, H3K4 methylation and increased glycolysis (46, 86). The same, sensing by TLR and signaling *via* mTOR pathways are involved in TRIM in vascular smooth muscle cells (48). Potentially this explains why training effects by oxLDL have been shown for non-myeloid tissues that also express the same receptors. In fact, this should also hold true for the other mediators of TRIM, but this, to our knowledge, has not been addressed experimentally

OxLDL may, however, also be considered in light of the recently suggested concept of LAMPs, which refers to molecules not associated with pathogens or cellular damage but instead arising from “failure-to-adapt-disease” such as observed in the context of atherosclerosis or gout. Key features of LAMPs have been defined as being persistent and having the ability to induce chronic disease (42). Furthermore, oxLDL cannot be cleared by the immune system and consequently induce chronic inflammation (30). We consider this potentially relevant for this topic as acute oxLDL exposure induces TRIM (46, 49), while LAMP-induced TRIM would involve a non-resolved stimulus and persistent activation, with the associated pro-inflammatory phenotype in phagocytes. Whether the observed link between high-fat diet, the predominant factor for the development of atherosclerosis, NLRP-3 inflammasome-dependent induction of TRIM in mice (87) is also mediated by oxLDL signaling is currently unclear.

### Vimentin

Vimentin is an intermediate filament protein involved in inflammatory responses and in Mφ endocytosis (88). Vimentin is a classical alarmin, sensed by Dectin-1 (38). While investigating donor allografts in a model of heart transplantation Braza et al. showed in the *ex vivo* second hit model, that isolated Mφ exposed to first vimentin and subsequent to HMGB-1 had an enhanced cytokine release of TNF and IL-6 (45). HMGB-1 is a DNA chaperone that mainly signals *via* extracellular receptor for advanced glycation end products (RAGE), a DAMP-specific receptor that also recognized S100 members and TLR-4 (89). This experimental set-up detaches the phenomenon of TRIM from any pathogen and clearly links it to sterile inflammation (30). However, as a limitation the authors here only provide direct *ex vivo* data and do not show whether each single component or a switched cadence would equally result in enhanced cytokine release. Of note, data using isolated splenocytes that were incubated first with HMGB-1 for eight days and then subsequently stimulated with LPS had a six-fold increase of TNF release in contrast to non-HMGB-1 exposed cells (90). While in this setting it is possible that HMGB-1 provides a continuous stimulation, the long protocol also could suggest that HMGB-1 can act as a trainer.

In this review, we describe DAMPs for which it was experimentally shown that they induce innate immune training. We also want to highlight, that at least in our hands, exposure to certain other DAMPs does not lead to trained immunity. Potentially, this was also observed in other laboratories but not reported. This could indicate shared characteristic of those DAMPs that induce TRIM, which still have to be identified. Exemplarily, we were not able to induce innate immune training with the short-lived ATP that binds to P2YR and P2XR and provokes immune activation in other models (91). The lack of ATP-induced TRIM might be explained by the fact that ATP does not bind the classical PRRs in contrast to the TRIM-inducing DAMPs. Furthermore, it is unclear, at least in the *in vitro* models, whether Mφ can clear TRIM-inducing DAMPs or their degradation products, whereas ATP for example is rapidly used by the cell and cleared (91).

## Does Damp Trim Fit Into the Two Signal Framework for Immune Activation?

In his introduction to the *Cold Spring Harbour Symposium* in 1989 Charles Janeway introduced the idea of PRR and the necessity of a two-signal system for immune activation (31). In subsequent work he proposed that a danger signal from the host is in fact a co-stimulation for the host that can act additionally to the classical pathogen-derived co-stimulation providing the needed signal two (92). While activation of adaptive immunity requires signals from two cells, in the evolutionarily older innate immune cell, no such co-stimulation existed and a meaningful second signal could have come from a different endogenous source. This could be the evolutionary justification for the observed phenomenon of innate immune memory. The changes in the bone marrow might be the reflection of the long-term consequences as cellular memory might evolutionarily not have yet been possible due to a lack of adaptive immunity. Why some DAMPs can act as signal one while others do not, remains to be established.

## Concluding Remarks

Over the last 15 years it has become clear that memory is a general feature of innate immunity. Strikingly, DAMP- and PAMP-induced trained immunity show comparable molecular reaction pattern. Recognition of both induce histone modifications and long-term persistent alteration of myelopoiesis that impact on the immune response upon secondary stimulation. This coherence hints towards an evolutionary conserved program, with logical advantages and so far, not understood disadvantages for the host mounting a secondary inflammatory program. Yet, it remains unclear under which conditions it is beneficial and when it is deleterious. This needs to be addressed for future application of the TRIM concept especially if applied in clinical settings.

Several further questions remain to us. Why are certain DAMPs worth remembering while others apparently not? How does DAMP-induced TRIM affect leukocyte trafficking, adaptive immunity, iNKT cell regulation and repair? Especially as damage signaling should result in the induction of a repair response. Is there an intracellular signaling funnel *via* which this reaction pattern is transmitted or can only DAMPs that can activate extracellular PRR-signaling lead to innate immune training? If intracellular PRR recognize DAMPs and initiate innate immune training, how would a constant immune activation be prevented? And ultimately does an epigenetic imprinting in the myeloid compartment have an evolutionary advantage to defend against pathogens? We are confident that the next years will shed light on some of these questions.

## Author Contributions

EJ and SW wrote the manuscript. All authors contributed to the article and approved the submitted version.

## Funding

There was no specific funding for this review. EJ is currently funded by the Deutsche Forschungsgemeinschaft (DFG, German Research Foundation) under Germany´s Excellence Strategy – EXC 2051 – Project-ID 390713860. SW is currently funded by the Deutsche Forschungsgemeinschaft, DFG, project number WE 4971/6-1 and The Federal Ministry of Education and Research (BMBF) project number 01EN2001.

## Conflict of Interest

The authors declare that the research was conducted in the absence of any commercial or financial relationships that could be construed as a potential conflict of interest.

## Publisher’s Note

All claims expressed in this article are solely those of the authors and do not necessarily represent those of their affiliated organizations, or those of the publisher, the editors and the reviewers. Any product that may be evaluated in this article, or claim that may be made by its manufacturer, is not guaranteed or endorsed by the publisher.
